# *CTLA4* Variants and Haplotype Contribute Genetic Susceptibility to Myasthenia Gravis in Northern Chinese Population

**DOI:** 10.1371/journal.pone.0101986

**Published:** 2014-07-08

**Authors:** Liang Sun, Yunxiao Meng, Yanchen Xie, Hua Zhang, Zheng Zhang, Xiaoxia Wang, Bin Jiang, Wei Li, Yao Li, Ze Yang

**Affiliations:** 1 The key Laboratory of Geriatrics, Beijing Hospital & Beijing Institute of Geriatrics, Ministry of Health, Beijing, China; 2 Department of Pathology, Peking Union Medical College Hospital, Chinese Academy of Medical Science, Tsinghua University, Beijing, China; 3 Department of Neurology, Beijing Friendship Hospital, Capital Medical University, Beijing, China; 4 Department of Neurology, Beijing Hospital, Ministry of Health, Beijing, China; 5 Department of Public Health, Ningxia Medical University, Yinchuan, China; Charité Universitaetsmedizin Berlin, Germany

## Abstract

**Background:**

Cytotoxic T lymphocyte-associated antigen-4 (CTLA4), a critical negative regulator of the T-cell response, has been considered a candidate for many autoimmune diseases. Evidence from Caucasians supported a genetic predisposition of *CTLA4* to myasthenia gravis (MG), but the contribution in East Asians has not been established.

**Objectives:**

To investigate the role of *CTLA4* variants in the susceptibility to MG and the contribution to subtypes of MG.

**Methods:**

Six autoimmune disease-related risk alleles of *CTLA4* (rs1863800, rs733618, rs4553808, rs5742909, rs231775, and rs3087243) were investigated for MG in northern Chinese. 168 patients with MG (mean age 37.1±20.5 years, 64 men and 104 women) and 233 healthy controls (mean age 53.3±8.7 years, 96 men and 137 women) were screened, and the contribution of *CTLA4* to the general risk of MG and each subgroup was explored.

**Results:**

rs1863800*C, rs733618*C, and rs231775*G were significantly associated with the whole cohort of patients with MG after permutation correction for multiple-testing adjustment (*P* = 0.027, 0.001, and 0.032, respectively). A risk haplotype (CCACG) [odds ratio (OR) = 1.535, range = 1.150–2.059, *P* = 0.004)] was also identified. The stratified subtype analysis indicated that the positive contribution was possibly derived from early onset MG (EOMG), seropositive MG (SPMG), female patients, and MG without thymoma. No association was observed in juvenile MG/LOMG, and MG coupled with thymoma.

**Conclusion:**

A predisposing effect of rs1863800*C, rs733618*C, and rs231775*G of *CTLA4* gene to general risk of MG in Chinese was demonstrated for the first time, which was likely derived from EOMG, SPMG, MG without thymoma and the female patients.

## Introduction

Myasthenia gravis (MG) is the most common neuromuscular junction disorder, characterized by fatigue and weakness of the striated muscles [1]. Because of the improved diagnosis and increasing lifespan, the overall prevalence of MG has increased over time with recent estimates approaching 20 per 100,000 in the US. In some other large populations, epidemiological surveys are still incomplete (e.g. in China and sub-Saharan Africa). Many patients experience intermittent worsening of symptoms triggered by infections, emotional stress, surgeries, or medications, particularly within the first 2 years of onset [2]. When bulbar or respiratory muscles are involved, MG could also be life-threatening.

Because of the complex clinical associated features, MG has been classified into subtypes based on muscles involved (ocular/generalized), age at onset, thymic abnormalities and autoantibody profiles. Approximately 10–15% cases of MG accompany thymoma. There are two major subgroups, early onset MG (EOMG) and late onset MG (LOMG), according to age at onset of MG. Most studies considered the 50 years at onset as the cut off value [3]. Typically, the cases of EOMG present a strong female preponderance [4]. LOMG might be more heterogeneous than EOMG, since the longer term of potential interaction between genetic and environmental factors. In addition, 80–85% of cases of MG are caused by autoantibodies against muscle acetylcholine receptor (AChR) [5].

The etiology of MG is complex which could be explained by a combination of genetic and unknown environmental factors [6]. However, the precise origin of the autoimmune response in MG is unknown. High incidence of thymic abnormalities strongly suggests a role for thymus in the process of MG [7]. In the abnormal status of thymus, aberrant negative selection may permit autoreactive T-cells to persist. Alternatively, aberrant positive selection may produce novel autoreactive T-cell [8]. Thus, T-cell-dependent B-cell activation of autoantibodies is likely to be crucial in the pathogenesis of MG [9].

The human leukocyte antigen (HLA) complex is implicated as a major genetic risk factor in many immune-mediated diseases [10]; however, its genetic predisposition is neither sufficient nor necessary for development of disease [11]. Cytotoxic T lymphocyte associated antigen-4 [CTLA4; cluster of differentiation (CD152)] is another underlying non-HLA candidate in autoimmune diseases, including MG [4], [12]. As a vital negative regulator for activation of T-cell [13], CTLA4 could competitively interfere with the binding of CD28 to B7-1 and B7-2 on antigen-presenting cells [14]. The CTLA4 knockout mouse exhibits a profound spontaneous autoimmune disease [15]. Together these observations suggest that CTLA4 might play a critical role in regulating self-tolerance, and hence in susceptibility to autoimmune disease.

The human *CTLA4* maps to chromosome 2q33. Several variants of *CTLA4* have been extensively tested, indicating an overall influence on the susceptibility of several immune-related diseases [16]–[21]. Although CTLA4 expressed similarly between MG and control peripheral blood mononuclear cells, rs733618, and rs4553808 could influence the *CTLA4* mRNA level [22]. In addition, rs733618, and rs4553808 were reported to be associated with MG by influencing the alternative splicing and expression of *CTLA4* in Swedish-Caucasians [23]; rs231775 was associated with thymoma manifestations of MG in Swedish-Caucasians and German-Caucasians [23], [24]. Although the association with MG and related subtypes has been re-evaluated in more cohesive groups of patients, the contribution in East Asians has not been established.

Accordingly, a comprehensive genotyping of six previously identified autoimmune-related candidate variants in *CTLA4* (rs1863800, rs733618, rs4553808, rs5742909, rs231775, and rs3087243) was carried out as the largest study in Chinese patients with MG by far. The study mainly focused on the general role of *CTLA4* variants in the susceptibility to MG. Secondarily, the clues about the contribution to subtypes of MG were further investigated.

## Materials and Methods

### Ethical approval of the research protocol

The Ethics Committees of Beijing Friendship Hospital, Capital Medical University and Beijing Hospital, Ministry of Health approved the study protocol. All participants were informed and provided informed consent in writing. All clinical investigation has been conducted according to the principles in the Declarations of Helsinki.

### Subject population

A total of 168 unrelated patients with MG (mean age 37.1±20.5 years, 64 men and 104 women) were included in the study. They were enrolled in Beijing friendship hospital, Capital Medical University and fulfilled the clinical and electromyography diagnostic criteria for acquired MG. Information on age at onset, AChR and MuSK status (ELISA kit), thymus status, involved muscles and Osserman type during 2 years follow-up were obtained. The sub-classification of patients was done according to age at onset, muscle involvement, status of AChR and MuSK antibodies, status of thymoma and gender (Table 1). The geography and ethnically matched control group consisted of 233 unrelated healthy subjects (mean age 53.3±8.7 years, 96 men and 137 women).The same control sample set has been used in other studies [25]. All the subjects studied were northern Chinese.

**Table 1:** Clinical Characteristics of 168 patients with MG.

Variables	n (male/female)
n	168 (64/104)
Age at MG onset	
<15 years (JMG)	37 (19/18)
15–50 years (EOMG)	90 (34/56)
≥50 years (LOMG)	41 (11/30)
AChR/MuSK antibody status	
SPMG	103 (38/65)
SNMG	51 (23/28)
Thymus status	
Thymoma	28 (15/13)
Non-thymoma	140 (49/91)
Osserman type during 2 years follow-up	
I	69 (27/42)
IIa	54 (20/34)
IIb	31 (11/20)
III	10 (6/4)
IV	4 (0/4)

MG = myasthenia gravis; AChR = acetylcholine receptor; JMG = juvenile MG; EOMG = early-onset MG; LOMG = late-onset MG; SPMG = Seropositive MG; SNMG = Seronegative MG;

### Genotyping

The genomic DNA was extracted from peripheral blood leukocytes using a standardized salting-out procedure. The six variants in *CTLA4* (rs1863800, rs733618, rs4553808, rs5742909, rs231775, rs3087243) were identified following polymerase chain reaction based restriction fragment length polymorphism (PCR-RFLP). Briefly, genomic DNA was amplified by PCR in PTC-225 (MJ RESEARCH, USA), using designed primers. Optimum PCR amplification was achieved with 1×PCR buffer, 2 mM MgCl2, 0.15 µM of each primer, 0.2 mM dNTP, 1.0 Unit Taq polymerase and 20 ng genomic DNA. (Table S1). The accuracy of genotyping has been further confirmed by Sanger's sequencing in 30 randomly selected cases.

### Statistical analyses for the case-control study

Allele frequencies were determined by gene counting and the fit for Hardy-Weinberg equilibrium (HWE) was verified using Chi-square goodness-fit test. Multiple logistic regression analysis was used to test the allele risk contribution under each genetic model. SNPStats program was used for test the allele association under additive, dominant, recessive and log-additive models [26].And the odds ratio (OR) was used to estimate the strength of association between variables, with the OR 95% confidence intervals. The HapMap genotype data (HCB/CEU) was searched from the official homepage (http://hapmap.ncbi.nlm.nih.gov/). Haploview (version 4.2) was used to estimate the linkage disequilibrium (LD) and haplotype among genotyped variants. Value for D′ and r^2^ were calculated for allele combination. Only common haplotypes with an estimated frequency >1% were considered. Permutation test (n = 1000) was conducted for the multiple-testing adjustment. Two-sided P<0.05 was considered statistically significant. Power calculations were performed with the program Genetic Power Calculator [27]. ROC_AUC_ and population attributable risk (PAR) was estimated for risk alleles that remained significant after adjustment using Statistical Package for Social Sciences (SPSS, version 12.0).

## Results

**Figure 1:**
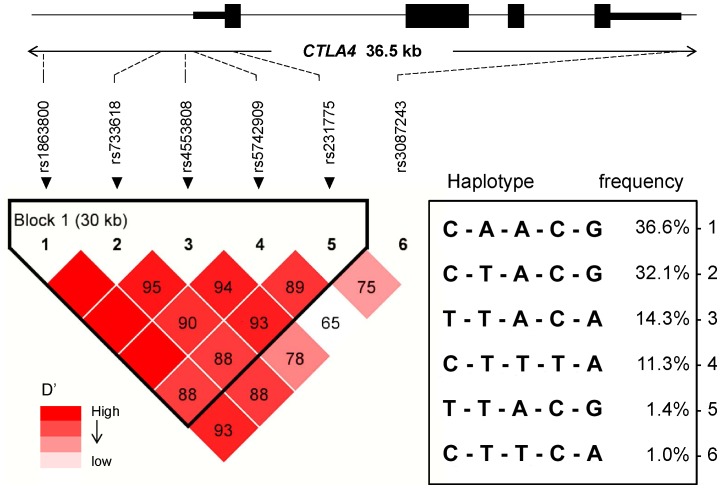
Haplotype block and LD test of candidate *CTLA4* variants in 36.5 kb genomic region. Generated by Haploview (version 4.2), and each box represents the D′ valuebetween pairs of SNPs (ranging from 0 to 1). Dark red, strong LD; light red, weak LD.

The six variants spanned 36.5 kb of genomic region (Figure 1). All of them were successfully genotyped and fitted the Hardy-Weinberg equilibrium. Compared to those in HapMap CEU, the minor allele frequencies (MAFs) of rs1863800, rs231775, and rs3087243 were much lower, and the MAFs of rs733618 and rs5742909 were much higher (Table 2). The power analysis showed that 94.9% (additive) and 89.3% (allelic) power were required to detect a genotype relative risk of 2.5 at an alpha level of 0.05 for variants with an MAF of 30%, assuming a prevalence of 0.01% of MG in Chinese.

**Table 2:** Summary of information of candidate SNPs in *CTLA4* gene.

SNPs	Aliases	allele^a^	Position^b^	Function	HWE^c^	MAF^d^	MAF_HapMap_ e
rs1863800	NA	C/T	204702660	5′-upstream	0.67	0.12/0.19	0.27/0.48
rs733618	−1722	T/C	204730944	promoter	0.97	0.45/0.33	0.38/0.06
rs4553808	−1661	A/G	204731005	promoter	0.61	0.11/0.15	0.08/0.11
rs5742909	−318	C/T	204732347	promoter	0.43	0.10/0.14	0.12/0.08
rs231775	+49/T17A	G/A	204732714	missense	0.55	0.24/0.32	0.31/0.61
rs3087243	CT60	G/A	204738919	3′-downstream	0.34	0.15/0.20	0.19/0.46

a Alleles underlined were the minor alleles identified in this study;

b Position based on the GRCh37/hg19 (Chromosome 2);

c *P* value of Hardy-Weinberg test;

d MAF of this study (MG/control);

e MAF of HapMap genotype (HCB/CEU); NA = not available.; MAF = minor allele frequency.

Analysis of pairwise LD among the six variants showed that the first five variants existed tightly in one block (*P*<0.05, D′>0.85) (Table S2). A total of six common haplotypes were identified across the LD block, ranging in frequency from 36.6 to 1% in total subjects (Table 3). One risk haplotype (CCACG) and one protective haplotype (TTACA) were identified after permutation correction for multiple-testing adjustment [odds ratio (OR) = 1.535, range = 1.150–2.059, *P* = 0.004 and OR = 0.543, range = 0.354–0.834, *P* = 0.030, respectively).

**Table 3:** Haplotype analysis of the *CTLA4* gene variants between MG and control subjects.

ID	Haplotype^a^	Frequency		χ^2^	*P* b	*P* c	OR (95%CI)
		MG	Controls				
1	C-C-A-C-G	0.423	0.325	8.440	0.004	0.021	1.535 (1.150–2.059)
2	C-T-A-C-G	0.306	0.332	0.667	0.414	0.993	0.882 (0.653–1.192)
3	T-T-A-C-A	0.101	0.173	7.954	0.005	0.030	0.543 (0.354–0.834)
4	C-T-T-T-A	0.080	0.138	6.302	0.012	0.068	0.549 (0.342–0.881)^c^
5	T-T-A-C-G	0.015	0.012	0.058	0.810	1.000	1.158 (0.350–3.827)^c^
6	C-T-T-C-A	0.021	0.003	9.794	0.002	0.071	2.416 (2.225–2.625)^c^

a haplotypes constructed by rs1863800-rs733618-rs4553808-rs5742909-rs231775;

b raw *P* value compared between groups;

c *P* value by permutation (n = 1000) correction; c statistical power is not enough (<70%).

Considering the rarity of MG and limited sample size in subgroups, the association between individual variants and general risk of MG was mainly focused (Table 4). The additive, log-additive and allele contrast model all indicated that the minor alleles of rs1863800, rs733618, rs231775, and rs3087243 distributed differently between MG and controls. After a further permutation correction (n = 1000) for multiple-testing adjustment, only rs1863800, rs733618, and rs231775 reached significance (*P* = 0.027, 0.001 and 0.032, respectively). The subsequent multifactorial logistic regression suggested that rs1863800*C, rs733618*C, and rs231775*G could increase the general risk of MG after filtering the confounding factors. After stratification by gender, it was found that all those three risk alleles associated with MG were significant in females (Table 5). The estimated PARs for MG were 40.42%, 17.96% and 28.50% for rs1863800*C, rs733618*C, and rs231775*G, respectively. The specificity and sensitivity of the regression model were calculated by constructing receiver operating-characteristic (ROC) curves, and the area under the curve (AUC) was calculated to estimate the ability of each risk variants to distinguish case subjects from control subjects. The combined three risk alleles resulted in an ROC_AUC_ value of 0.669 (Table 6).

**Table 4:** Distribution and compare of genotypes and alleles of candidate variants between MG and control subjects.

variants	Genotype	MG	Control	*P*_additive_	*P*_log-additive_	*P*_allele_	*P*_permutation_ a
rs1863800	CC	133	152	0.008	0.005	0.005	0.027
	CT	31	74				
	TT	4	7				
rs733618	TT	62	105	<0.001	0.001	<0.001	0.001
	TC	61	103				
	CC	45	25				
rs4553808	AA	135	169	0.180	0.110	0.099	0.558
	AG	29	58				
	GG	4	6				
rs5742909	CC	138	172	0.160	0.067	0.062	0.340
	CT	27	54				
	TT	3	6				
rs231775	GG	98	104	0.022	0.007	0.008	0.032
	AG	60	107				
	AA	10	22				
rs3087243	GG	125	146	0.030	0.045	0.046	0.288
	GA	37	79				
	AA	6	7				

a *P* value by permutation (n = 1000) correction based on allele contrast model.

**Table 5:** Association analysis of risk allele of *CTLA4* variants with risk of MG under each multi-factorial model.

models	rs1863800*C	rs733618*C	rs231775*G
model 1^b^			
additive	1.75 (1.18–2.63)^a^	1.58 (1.20–2.08)^a^	1.54 (1.12–2.13)^a^
dominant	2.04 (1.28–3.23)^a^	1.40 (0.93–2.10)	1.72 (1.16–2.56)^a^
recessive	1.27 (0.37–4.35)	3.04 (1.78–5.21)^a^	1.64 (0.76–3.57)
model 2^c^			
additive	1.72 (1.12–2.70)^a^	1.58 (1.17–2.14)^a^	1.53 (1.08–2.17)^a^
dominant	1.96 (1.19–3.23)^a^	1.42 (0.91–2.20)	1.72 (1.11–2.63)^a^
recessive	1.32 (0.34–5.26)	2.92 (1.65–5.16)^a^	1.61 (0.69–3.70)
model 3^d^			
male	2.17 (0.85–5.56)	2.36 (0.88–6.34)	1.28 (0.59–2.78)
female	1.67 (1.04–2.63)^a^	2.67 (1.20–5.94)^a^	2.04 (1.14–3.57)^a^

a *P* value<0.05;

b model 1 (crude estimation);

c model 2 (filtered the juvenile MG);

d model 3 (based on the previous most significant genetic model, stratified by gender, filtered the juvenile MG, and adjusted by age).

**Table 6:** ROC_AUC_ values calculation with three identified *CTLA4* risk alleles.

Variants ID	risk alleles^a^	ROC_AUC_ values^b^	95% CI
rs1863800	C	0.570	0.513–0.626
rs733618	C	0.580	0.523–0.638
rs231775	G	0.568	0.512–0.625
Combined	C-C-G	0.669	0.585–0.753

a based on the corresponding genetic models in **Table 4**;

b ROC_AUC_: the area under receiver operating characteristics (ROC) curve.

**Figure 2:**
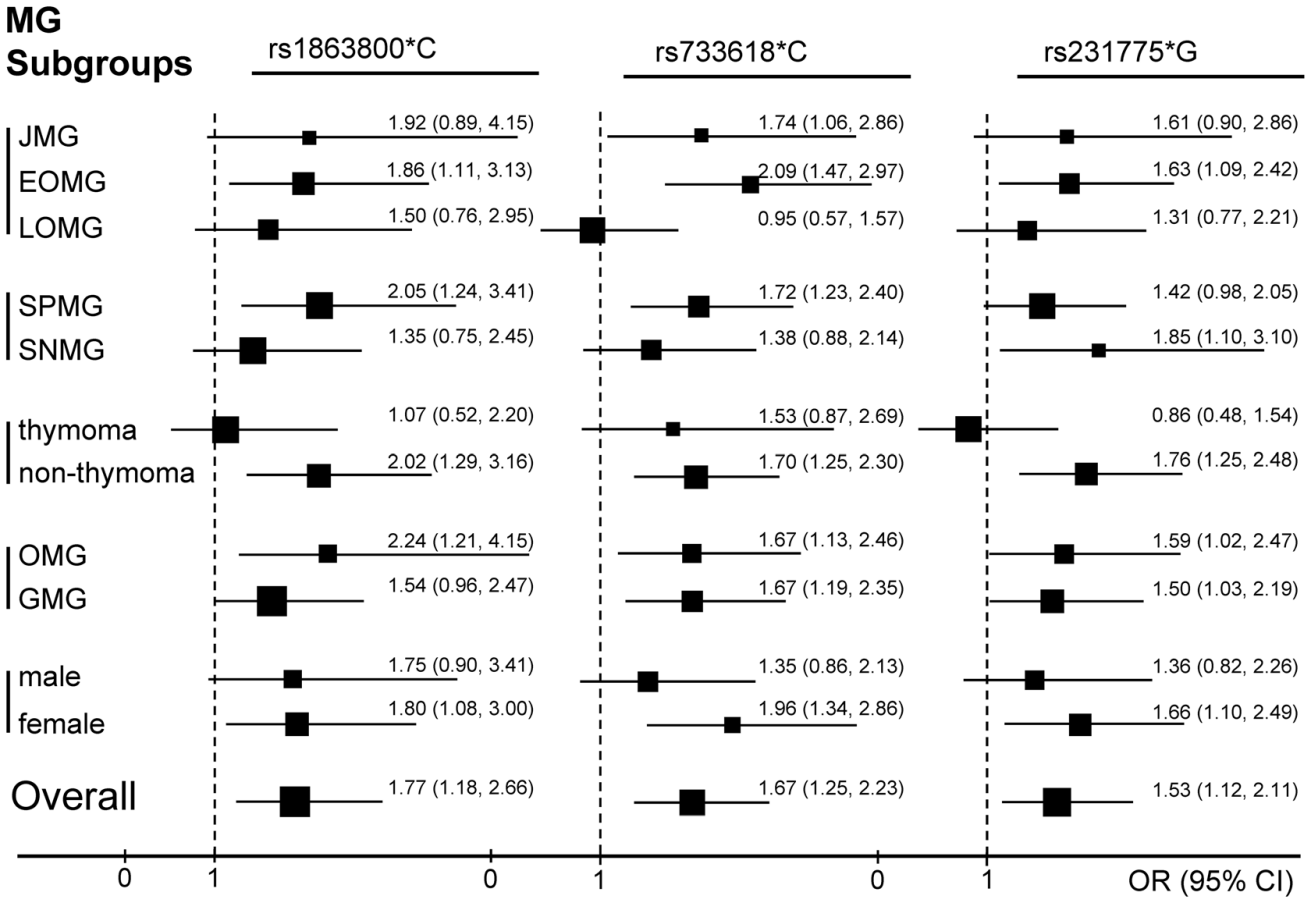
Odds ratios and 95% confidence intervals (CI) of 3 *CTLA4* risk alleles with MG subgroups. JMG = juvenile MG; EOMG = early-onset MG; LOMG = late-onset MG; SPMG = Seropositive MG; SNMG = Seronegative MG; OMG = ocular myasthenia gravis; GMG = generalized myasthenia gravis.

In addition, a stratified subtype analysis was conducted between the three identified risk alleles and each MG subtype based on the allele contrast model (Figure 2) (Table S3). On the whole, the contribution of rs1863800*C, rs733618*C, and rs231775*G for MG existed more significantly in EOMG, seropositive MG (SPMG), female patients, and MG without thymoma than the respective opposite subgroups. rs733618*C was critically associated with juvenile MG (JMG). However, none of these above alleles were associated with LOMG and those coupled with thymoma. Both ocular and generalized MG was associated with all the three risk alleles.

Moreover, the distribution of above three risk alleles among subgroups were compared only within the patients with MG according to the age at onset of MG, AChR/MuSK antibody status, thymus status, muscles involved, Osserman type and gender, where an increased 733618*C in EOMG than in LOMG (0.506 vs 0.317) and an increased rs231775*A in patients with thymoma than those without thymoma (0.357 vs 0.214, OR = 2.037, range = 1.099–3.774) were observed (Table S3).

## Discussion

To our knowledge, this is the first report of the genetic contribution of *CTLA4* to MG in East Asians. Overall, rs1863800*C, rs733618*C, and rs231775*G could confer the general risk of MG, even after the permutation correction and adjustment for covariance. A haplotype CCACG, containing rs1863800*C, rs733618*C, and rs231775*G was identified to increase the general risk of MG by 1.535-fold (*P* = 0.021, after permutation adjustment). The stratified subtype analysis indicated that the positive contribution was likely derived from EOMG, SPMG, female patients, and patients with MG without thymoma. In addition, the case-only analysis indicated that rs733618*C and rs231775*A were also associated with EOMG and presence of thymoma, respectively. These findings are consistent with a role of *CTLA4* variants in predisposition to MG.

There are accumulating evidences to suggest the role of *CTLA4* variants to autoimmune diseases including systemic lupus erythematosus (SLE), rheumatoid arthritis (RA), Graves' disease, Hashimoto's thyroiditis, postpartum thyroiditis, Addison's disease, insulin-dependent diabetes mellitus, vitiligo and multiple sclerosis [28]–[36]. With regard to MG, the most significant association was reported for two promoter variants (rs733618 and rs4553808) in Swedish-Caucasians [23]. Besides, rs231775*A, which was protective against several autoimmune diseases, was reported to exert a predisposing effect to paraneoplastic MG in thymoma German-Caucasian patients [24]. Recently, the same research group reported an opposite result that rs231775*G was associated with LOMG in German-Caucasians [37].

The genetic structures are obviously different among racial/ethnic groups, resulting in diverse genetic susceptibilities to a certain disease [38]. Many of the studies indicated that MG in East Asians might be clinically different from that in Caucasians [39], [40]. The pediatric patients, mainly with purely ocular MG, are more frequent in the East Asians than Caucasians. In addition, the HLA complex also influenced the risk of MG differently between East Asians and Caucasians [41]–[43], suggesting that etiological differences might exist among ethnicities [44]. However, no study was conducted in East Asians so far. Because of the rarity of MG, the previous several available reports were almost based on 50–150 patients with MG. Although the clinical heterogeneity is serious, the stratified subtype analysis would decrease the statistical power definitely. Accordingly, the contribution of *CTLA4* to the general risk of MG with an improved power was mainly wished to be determined.

Gender is the main confounding factor in most of the autoimmune diseases [45], [46]. Data from the present study suggested that females with risk alleles might be more predisposed to MG than males. However, nearly all the reports about *CTLA4* and MG were neither based on gender-matched subjects nor stratified analysis performed to remove the bias. This might be due to the decreased statistical power for the fewer males in the present study, because the trend of contribution existed, although it did not reach the significant level. Or, *CTLA4* variants played indeed different roles of MG between genders in accordance with diversity in clinical manifestations between genders.

The subtype analysis also indicated that *CTLA4* risk alleles mainly confer the risk of EOMG. Both the studies in Caucasians did not discuss the differences in *CTLA4* among EOMG, LOMG and JMG [47], [48]. Interestingly, a recently study in German-Caucasians suggested rs231775*G was associated with LOMG, where EOMG was not discussed [37]. Complementarily, it was found that the rs231775*G was associated with EOMG rather than JMG and LOMG. This might be related to “≥60 years” as the cutoff value for LOMG differed from “≥50 years” in the present study. When the criteria was updated to “≥60 years” in the present study, only 28 subjects were included presenting no association with LOMG yet. A reasonable explanation was that there might be more environmental risk factors and aging involved before the onset of LOMG than EOMG; accordingly, the mechanism for LOMG could be more heterogeneously coupled with ocular or generalized weakness, typically have a more severe disease course compared with EOMG [4].

In addition, the contribution of *CTLA4* to MG was dependent on non-thymoma status, indicating a unique pathogenesis of paraneoplastic MG, in accordance with the previous report in Caucasians [24]. The case-only analysis in the present study indicated that rs231775*A was also associated with MG coupled with thymoma (OR = 2.037, range = 1.099–3.774), which is opposite to its protective role reported in other autoimmune diseases. The paradox that the gain-of-function rs231775*A in predisposing to paraneoplastic MG could be explained by the nontolerogenic selection of CD4^+^ T-cells in MG-associated thymomas [24].

Because of the evidence that pathogenesis of MG might be different between SPMG and seronegative MG [49], in the present study, the subjects were stratified by the serum antibody status and found that the contribution of three *CTLA4* risk alleles only existed in the SPMG subtype.

Since rs1863800*C, rs733618*C, and rs231775*G were linked tightly in a block (Figure 1), and no obvious accumulative effect was detected from the haplotype and combined ROC_AUC_ value, the three risk alleles might not work independently. Promoter analysis indicated that rs733618*C might disturb the binding with transcript factor, NF-1, which was validated by chromatin immunoprecipitation assay and gel shift assay [23]. rs231775*A (aliases: +49, T17A) is a gain-of-function missense mutation associated with altered expression and activation of T-cell [50], [51]. CTLA4 could exert the protective effect by attenuating the interaction of autoreactive T-cells and antigen-presenting cells in the peripheral immune system. Considering the tight linkage of three risk alleles, rs1863800*C-rs733618*C-rs231775*G might be corresponding to a lower surface expression of CTLA4 and reduced inhibitory function of CTLA4, predisposing to MG without thymoma. On the contrary, the contribution of thymoma to the development of paraneoplastic MG is nontolerogenic thymopoiesis inside the thymoma, i.e., central tolerance failure. The thymoma could promote intratumorous T-cell maturation to the CD4^+^ CD45RA^+^ naïve T cells. In addition, the positive association between *CTLA4* with MG could also be indirectly caused by relevant causal gene(s) in LD with *CTLA4* in East Asians. This should be prospectively explored by the large-scale annotation for ethnic-specific genome structure; whole-genome association studies (GWAS) and genome-sequencing for rare variants in larger and well-defined cohorts.

Some limitations should be addressed in the future. First, a more accurate and comprehensive diagnosis of each concomitant autoimmune components is necessary. Second, patients with non–MG thymoma should be included as another control group for better elucidation of roles of thymoma, MG and genetic determinants. Third, the gene-specific hypomethylation and noncoding RNAs are also potential mechanisms in many autoimmune diseases such as SLE and RA [52], [53], suggesting that the epigenetics of *CTLA4* might be addressed as well.

## Conclusions

The results from the present study demonstrate a predisposing effect of rs1863800*C, rs733618*C, and rs231775*G of *CTLA4* gene to general risk of MG in Chinese for the first time. In addition, the subtype analysis indicated that the contribution was likely derived from EOMG, SPMG, and MG without thymoma, and the female patients. Further validation in well-defined, larger sample size, different ethnic populations and GWAS are needed.

## Supporting Information

Table S1 Primers and genotyping conditions based on RFLP of candidate SNPs in *CTLA4* gene.
(DOCX)

Table S2 Linkage disequilibrium analysis of candidate *CTLA4* variants in MG and control subjects (D′/r^2^ statistic).
(DOCX)

Table S3 Description of the minor allele and genotype distribution of CTLA4 gene variants in each MG subgroups (%).
(DOCX)
