# Supplementary material for: CTLA4 Variants and Haplotype Contribute Genetic Susceptibility to Myasthenia Gravis in Northern Chinese Population
Source: PLoS One. 2014 Jul 8;9(7):e101986. doi: 10.1371/journal.pone.0101986 (PMC4086970; doi:10.1371/journal.pone.0101986)
Supplement: Table S1 — Primers and genotyping conditions based on RFLP of candidate SNPs in CTLA4 gene. (DOCX) [file pone.0101986.s001.docx]

**Table S1.** Primers and genotyping conditions based on RFLP of candidate variants in *CTLA4* gene

| SNPs |  | Primer sequence (5’-3’) | Tm  (℃) | Length | RFLP  enzyme | Digestion  Fragment (bp) |
| --- | --- | --- | --- | --- | --- | --- |
| rs1863800 | Forward | GCTAAAACCTAACAACAATCAAG | 56 | 87 | *Tas* I | T:63,24 |
|  | Reverse | CTAAGGCTTTTCTTTACTATCA |  |  |  |  |
| rs733618 | Forward | CTAAGAGCATCCGCTTGCACCT | 59 | 486 | *Bbv* I | C: 257,229 |
|  | Reverse | TTGGTGTGATGCACAGAAGCCTTTT |  |  |  |  |
| rs4553808 | Forward | CTAAGAGCATCCGCTTGCACCT | 59 | 486 | *Mse* I | A:347,139 |
|  | Reverse | TTGGTGTGATGCACAGAAGCCTTTT |  |  |  |  |
| rs5742909 | Forward | AAATGAATTGGACTGGATGGT | 61 | 247 | *Mse* I | T:21,96,130 |
|  | Reverse | TTACGAGAAAGGAAGCCGTG |  |  |  |  |
| rs231775 | Forward | CCACGGCTTCCTTTCTCGTA | 55.5 | 327 | *Bbv* I | G:244,84 |
|  | Reverse | AGTCTCACTCACCTTTGCAG |  |  |  |  |
| rs3087243 | Forward | ATGAGTCAGCTTTGCACCAGCCATTAC | 61 | 167 | *Nla* III | A:97,43,23,4 |
|  | Reverse | GAGGTGAAGAACCTGTGTTAAACAGCATG |  |  |  |  |
