# Supplementary material for: CTLA4 Variants and Haplotype Contribute Genetic Susceptibility to Myasthenia Gravis in Northern Chinese Population
Source: PLoS One. 2014 Jul 8;9(7):e101986. doi: 10.1371/journal.pone.0101986 (PMC4086970; doi:10.1371/journal.pone.0101986)
Supplement: Table S2 — Linkage disequilibrium analysis of candidate CTLA4 variants in MG and control subjects (D′/r2 statistic). (DOCX) [file pone.0101986.s002.docx]

**Table S2.** Linkage disequilibrium analysis of candidate *CTLA4* variants in MG and control subjects (D'/r^2^ statistic)

| variants | rs1863800 | rs733618 | rs4553808 | rs5742909 | rs231775 | rs3087243 |
| --- | --- | --- | --- | --- | --- | --- |
| rs1863800 | - | 0.999/0.115 | 0.998/0.029 | 0.997/0.026 | 0.883/0.362 | 0.932/0.757 |
| rs733618 | - | - | 0.955/0.086 | 0.905/0.071 | 0.886/0.194 | 0.888/0.104 |
| rs4553808 | - | - | - | 0.943/0.816 | 0.938/0.335 | 0.787/0.021 |
| rs5742909 | - | - | - | - | 0.899/0.282 | 0.653/0.013 |
| rs231775 | - | - | - | - | - | 0.755/0.304 |
| rs3087243 | - | - | - | - | - | - |
