# Supplementary material for: CTLA4 Variants and Haplotype Contribute Genetic Susceptibility to Myasthenia Gravis in Northern Chinese Population
Source: PLoS One. 2014 Jul 8;9(7):e101986. doi: 10.1371/journal.pone.0101986 (PMC4086970; doi:10.1371/journal.pone.0101986)
Supplement: Table S3 — Description of the minor allele and genotype distribution of CTLA4 gene variants in each MG subgroups (%). (DOCX) [file pone.0101986.s003.docx]

**Table S3.** Description of the minor allele and genotype distribution of *CTLA4* gene variants in each MG subgroups (%)

| Variants | MG_Subgroup1_ ^b^ | | | MG_Subgroup2_ ^c^ | | MG_Subgroup3_ ^d^ | | MG_Subgroup4_ ^e^ | | MG_Subgroup5_ ^f^ | | Control | | |
| --- | --- | --- | --- | --- | --- | --- | --- | --- | --- | --- | --- | --- | --- | --- |
|  | JMG | EOMG | LOMG | SPMG | SNMG | thymoma | non-thymoma | OMG | GMG | male | female | overall | male | female |
| rs1863800 |  |  |  |  |  |  |  |  |  |  |  |  |  |  |
| CC | 30 | 72 | 31 | 84 | 37 | 20 | 113 | 57 | 76 | 51 | 82 | 152 | 66 | 86 |
| CT | 6 | 16 | 9 | 17 | 13 | 6 | 25 | 11 | 20 | 12 | 19 | 74 | 26 | 48 |
| TT | 1 | 2 | 1 | 2 | 1 | 2 | 2 | 1 | 3 | 1 | 3 | 7 | 4 | 3 |
| MAF ^a^ | 0.108 | 0.111 | 0.134 | 0.102 | 0.147 | 0.179 | 0.104 | 0.094 | 0.131 | 0.109 | 0.12 | 0.189 | 0.177 | 0.197 |
| rs733618 |  |  |  |  |  |  |  |  |  |  |  |  |  |  |
| TT | 14 | 27 | 21 | 38 | 22 | 10 | 52 | 24 | 38 | 24 | 38 | 105 | 36 | 69 |
| TC | 12 | 35 | 14 | 36 | 17 | 12 | 49 | 28 | 33 | 22 | 39 | 103 | 47 | 56 |
| CC | 11 | 28 | 6 | 29 | 12 | 6 | 39 | 17 | 28 | 18 | 27 | 25 | 13 | 12 |
| MAF | 0.459 | 0.506 | 0.317 | 0.456 | 0.402 | 0.429 | 0.454 | 0.449 | 0.449 | 0.453 | 0.447 | 0.328 | 0.38 | 0.292 |
| rs4553808 |  |  |  |  |  |  |  |  |  |  |  |  |  |  |
| AA | 29 | 73 | 33 | 78 | 46 | 22 | 113 | 56 | 79 | 52 | 83 | 169 | 72 | 97 |
| AG | 8 | 15 | 6 | 23 | 3 | 6 | 23 | 12 | 17 | 11 | 18 | 58 | 19 | 39 |
| GG | 0 | 2 | 2 | 2 | 2 | 0 | 4 | 1 | 3 | 1 | 3 | 6 | 5 | 1 |
| MAF | 0.108 | 0.106 | 0.122 | 0.131 | 0.069 | 0.107 | 0.111 | 0.101 | 0.116 | 0.102 | 0.115 | 0.15 | 0.151 | 0.15 |
| rs5742909 |  |  |  |  |  |  |  |  |  |  |  |  |  |  |
| CC | 29 | 77 | 32 | 82 | 47 | 24 | 114 | 58 | 80 | 55 | 83 | 172 | 74 | 98 |
| CT | 8 | 12 | 7 | 20 | 2 | 4 | 23 | 10 | 17 | 8 | 19 | 54 | 16 | 38 |
| TT | 0 | 1 | 2 | 1 | 2 | 0 | 3 | 1 | 2 | 1 | 2 | 6 | 5 | 1 |
| MAF | 0.108 | 0.078 | 0.134 | 0.107 | 0.059 | 0.071 | 0.104 | 0.087 | 0.106 | 0.078 | 0.111 | 0.142 | 0.137 | 0.146 |
| rs231775 |  |  |  |  |  |  |  |  |  |  |  |  |  |  |
| GG | 22 | 52 | 24 | 56 | 33 | 11 | 87 | 40 | 58 | 36 | 62 | 104 | 48 | 56 |
| AG | 13 | 35 | 12 | 42 | 15 | 14 | 46 | 26 | 34 | 24 | 36 | 107 | 36 | 71 |
| AA | 2 | 3 | 5 | 5 | 3 | 3 | 7 | 3 | 7 | 4 | 6 | 22 | 12 | 10 |
| MAF | 0.23 | 0.228 | 0.268 | 0.252 | 0.206 | 0.357 | 0.214 | 0.232 | 0.242 | 0.25 | 0.231 | 0.324 | 0.313 | 0.332 |
| rs3087243 |  |  |  |  |  |  |  |  |  |  |  |  |  |  |
| GG | 28 | 67 | 30 | 80 | 36 | 19 | 106 | 56 | 69 | 49 | 76 | 146 | 65 | 81 |
| GA | 7 | 20 | 10 | 21 | 13 | 7 | 30 | 11 | 26 | 13 | 24 | 79 | 26 | 53 |
| AA | 2 | 3 | 1 | 2 | 2 | 2 | 4 | 2 | 4 | 2 | 4 | 7 | 5 | 2 |
| MAF | 0.149 | 0.144 | 0.146 | 0.121 | 0.167 | 0.196 | 0.136 | 0.109 | 0.172 | 0.133 | 0.154 | 0.2 | 0.188 | 0.21 |

MG=myasthenia gravis; AChR=acetylcholine receptor; JMG=juvenile MG; EOMG=early-onset MG; LOMG=late-onset MG; SPMG=Seropositive MG; SNMG= Seronegative MG; OMG=Ocular MG; GMG=Generalized MG

*^a^* MAF=minor allele frequency (rs1863800-T, rs733618-C, rs4553808-G, rs5742909-T, rs231775-A, rs3087243-A); *^b^* Subgroup by age at MG onset; *^c^* Subgroup by AChR/MuSK antibody status; *^d^* Subgroup by thymoma status; *^e^* Subgroup by involved muscles
